# Pairagon+N-SCAN_EST: a model-based gene annotation pipeline

**DOI:** 10.1186/gb-2006-7-s1-s5

**Published:** 2006-08-07

**Authors:** Manimozhiyan Arumugam, Chaochun Wei, Randall H Brown, Michael R Brent

**Affiliations:** 1 Laboratory for Computational Genomics and Department of Computer Science, Washington University, One Brookings Drive, St. Louis, MO 63130, USA

## Abstract

**Background:**

This paper describes Pairagon+N-SCAN_EST, a gene annotation pipeline that uses only native alignments. For each expressed sequence it chooses the best genomic alignment. Systems like ENSEMBL and ExoGean rely on *trans *alignments, in which expressed sequences are aligned to the genomic loci of putative homologs. *Trans *alignments contain a high proportion of mismatches, gaps, and/or apparently unspliceable introns, compared to alignments of cDNA sequences to their native loci. The Pairagon+N-SCAN_EST pipeline's first stage is Pairagon, a cDNA-to-genome alignment program based on a PairHMM probability model. This model relies on prior knowledge, such as the fact that introns must begin with GT, GC, or AT and end with AG or AC. It produces very precise alignments of high quality cDNA sequences. In the genomic regions between Pairagon's cDNA alignments, the pipeline combines EST alignments with de novo gene prediction by using N-SCAN_EST. N-SCAN_EST is based on a generalized HMM probability model augmented with a phylogenetic conservation model and EST alignments. It can predict complete transcripts by extending or merging EST alignments, but it can also predict genes in regions without EST alignments. Because they are based on probability models, both Pairagon and N-SCAN_EST can be trained automatically for new genomes and data sets.

**Results:**

On the ENCODE regions of the human genome, Pairagon+N-SCAN_EST was as accurate as any other system tested in the EGASP assessment, including ENSEMBL and ExoGean.

**Conclusion:**

With sufficient mRNA/EST evidence, genome annotation without trans alignments can compete successfully with systems like ENSEMBL and ExoGean, which use trans alignments.

## Background

There are three fundamental approaches to automated construction of exon-intron structure for protein-coding genes: **native alignment **- alignment of expressed sequences (including high quality cDNA sequences, expressed sequence tags (ESTs), and protein sequences) to the loci from which they were transcribed; **trans alignment **- non-native alignment of expressed sequences to loci that could potentially express similar sequences (can be within or between species); and **de novo **- prediction using the sequences of one or more genomes as the only inputs (no expressed sequences).

Native alignments of full insert, high quality cDNA sequences are the unquestioned gold standard in high-throughput annotation. However, even a concerted, high-budget effort to sequence cDNA libraries produces a full-open reading frame (ORF) sequence for only about 50% to 60% of loci in a mammalian genome [1]. Thus, trans alignments have played a key role in producing the most trusted genome predictions, including the ENSEMBL predictions (sometimes termed 'evidence based') that have been used in the first published analyses of many new genome sequences. Nonetheless, the evidence they provide for expression is circumstantial rather than direct - for example, the annotated genomic locus may represent a pseudogene derived from the true genomic source of the expressed sequence. Even when a trans alignment identifies a functional homologous gene locus, the alignments tend to be inaccurate in their details unless the expressed sequence is highly similar to the genomic sequence [2,3].

*De novo *predictions have always been viewed with some suspicion. This suspicion derives in part from the tendency of gene predictors developed in the 1990s to predict far too many false positive genes and exons. It may also result, in part, from the fact that one cannot point to the evidence supporting *de novo *predictions - a large ensemble of individually weak statistical patterns - the way one can point to a single expressed sequence. Nonetheless, statistical evidence is biological evidence, with a track record extending back to Gregor Mendel.

If *de novo *prediction were indeed inaccurate, relying heavily on trans alignments would make sense when analyzing a genome for which few EST or cDNA sequences are available. However, the rapidly increasing accuracy of *de novo *prediction and the large number of very high quality cDNA sequences available for human suggest the possibility that high quality annotations might be produced without using trans alignments. A system that does not use trans alignments might be more accurate than one that does, since all alignments would have near 100% identity. Even if its accuracy were merely equal to that of a system using trans alignments, the evidence supporting each prediction might be considered more direct.

To build an annotation pipeline without trans alignment, we combined a number of tools that have been recently developed in our lab. These tools include Pairagon, a cDNA-to-genome aligner, N-SCAN_EST [4], a multi-genome gene predictor capable of taking guidance from EST alignments, and PPFINDER [5], a program for eliminating pseudogenes from sets of predicted protein-coding genes.

### Pairagon uses a PairHMM to produce native cDNA alignments

**Figure 1:**
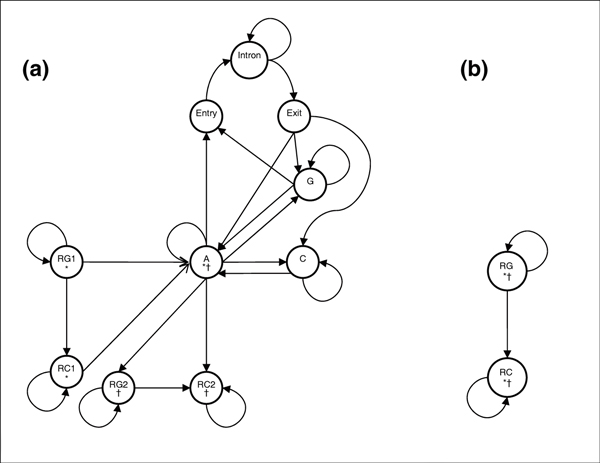
PairHMM state diagrams of Pairagon. **(a) **Alignment model and **(b) **Null model. RG1 and RG2 are unaligned genomic sequences in the 5' and 3' ends, respectively; RC1 and RC2 are unaligned cDNA sequences in the 5' and 3' ends, respectively; A, aligned; Entry corresponds to the first two bases of an intron; Exit corresponds to the last two bases of an intron; G, genomic insertion; C, cDNA insertion; RG and RC are random genomic and cDNA sequences, respectively. States that can start an alignment are marked with an asterisk and states that can end an alignment are marked with a dagger.

To produce the best possible alignments of high quality cDNA sequences, we used Pairagon, a cDNA-to-genome aligner that is based on a pairHMM probability model [6]. A pairHMM is a hidden Markov model (HMM) whose states emit alignment columns. In our case, the columns contain either a match between the two sequences, a mismatch, an insertion in the genome, a deletion in the genome, or an intron base in the genome (Figure 1). The particular pairHMM model we developed is 'strong', in the sense that it enforces prior biological and statistical knowledge rather than letting the data at hand dictate the alignment even when it is at odds with prior knowledge. In particular, our model only produces introns with plausible splice site sequences: GT-AG, GC-AG, AT-AC (AT-AG and other extremely rare U12 intron types [7] are not currently allowed). Furthermore, the probabilities of introns, matches, mismatches, genome insertions, and genome deletions are estimated from alignments of high quality cDNA sequences produced by BLAT [8] and the relative probabilities of the three intron types are derived from prior knowledge.

**Figure 2:**
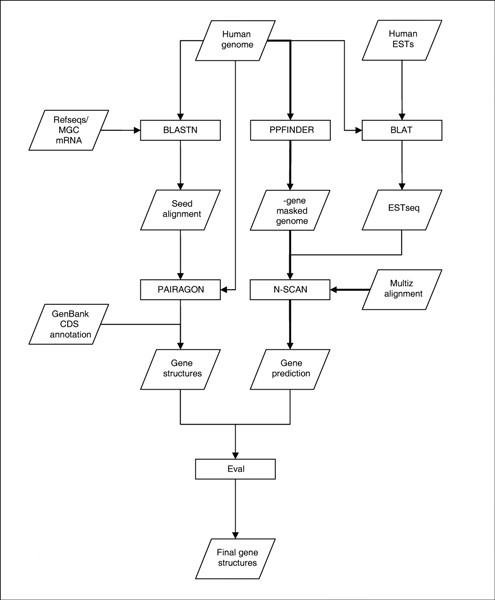
Block diagram of the Pairagon+N-SCAN_EST pipeline. The bold arrows mark the section of the flowchart corresponding to N-SCAN gene prediction.

In order to make Pairagon run faster, we ran ungapped BLASTN as a preprocessing step and used the long alignments it produced to seed exon alignments (Figure 2, left side). For more details on Pairagon and its heuristics, see Materials and methods.

Our strategy was to use alignments of expressed sequences directly only when very high quality sequences were available. Thus, we applied Pairagon only to full ORF Mammalian Gene Collection (MGC) sequences [1,9,10] and human RefSeq mRNAs [11].

### N-SCAN_EST threads complete gene structures through EST alignments

In the genomic regions between Pairagon's cDNA alignments, we combined EST alignments with *de novo *gene prediction by using N-SCAN_EST [4]. N-SCAN_EST is based on N-SCAN [12,13], a multi-genome *de novo *gene predictor, which was the most accurate *de novo *predictor in the EGASP assessment [14] by every measure except nucleotide sensitivity. (*De novo *includes both the '*ab initio*' and 'multi-genome' assessment categories.) N-SCAN_EST is a version of N-SCAN that takes guidance from EST alignments. Specifically, it takes as input a representation of EST alignments that we call ESTseq, by analogy to the 'conservation sequence' used in TWINSCAN (a three-character alphabet representing genome sequence conservation between two species) [15,16]. N-SCAN_EST takes guidance from EST alignments, but it does not follow them blindly. Instead, it also considers the DNA sequence of the target genome and the evolutionary conservation information provided by alignments of the target genome with the genomes of other organisms. It predicts complete transcripts by extending or merging EST alignments or by building gene structures in which some exon regions are supported by EST evidence while others are not. We have shown elsewhere that this approach increases sensitivity and specificity not only for the genes that have EST support, but even for those that do not [4].

### Pairagon+N-SCAN_EST annotates genomes without using *trans *alignment

To apply N-SCAN_EST, we downloaded human ESTs from dbEST and aligned them to the human genome using BLAT [8] (Figure 2, right side). We also downloaded alignments of the human, mouse, rat, and chicken genomes produced by MULTIZ [17] from the University of California Sant Cruz (UCSC) genome browser. These EST and genomic alignments were input to N-SCAN_EST. N-SCAN_EST was run on human genomic sequence that had been masked with PPFINDER, our processed pseudogene masker [5]. After the final round of N-SCAN_EST, all predicted transcripts that overlapped Pairagon alignments were removed. The remaining transcripts (one per locus) were combined with the Pairagon alignments to produce the final gene set.

In the remainder of this paper we present accuracy statistics for both the EGASP version of the pipeline and an updated version and analyze the relative contributions of Pairagon versus N-SCAN_EST. We then examine a series of examples where our pipeline gave a revealing result, whether correct or incorrect. Finally, we draw some lessons about how the pipeline could be improved in the future.

## Results and discussion

RefSeq and MGC cDNA sequences mapped to the ENCODE regions were downloaded from the UCSC Genome Browser and alignments were generated using the Stepping Stone implementation of Pairagon v0.5 as described in Materials and methods. GenBank's coding sequence (CDS) annotations of these cDNA sequences were used to produce 451 aligned transcripts annotated with GenBank ORFs (141 from MGC sequences and 310 from RefSeq sequences). Merging identical gene structures and removing inconsistent structures (for example, gap in the coding region leading to a frame shift in the genome) yielded 413 unique gene structures. N-SCAN_EST predictions were generated as described in Materials and methods. The 94 N-SCAN_EST predictions that did not overlap the 413 Pairagon gene structures were added to the gene set. We obtained seven gene structures by aligning sequences from our RT-PCR experiments. Two of these did not overlap the existing set and were included in our submission to the 'any evidence' category. We do not discuss this set in detail because it is almost identical to the submission to the 'mRNA/EST evidence' category. The accuracy statistics for this set can be found in the EGASP assessment report [14].

### The official assessment of Pairagon+N-SCAN_EST shows high accuracy

Table 1 compares the coding region prediction accuracy measures of three submissions to the EGASP 'mRNA/EST evidence' category at the gene, transcript, exon and nucleotide levels. Pairagon+N-SCAN_EST (Pairagon+N) is optimized for high accuracy in predicting exact exons and transcripts, so we will focus our analysis on those columns of Table 1. By both measures, ExoGean is the most sensitive of the three programs and Pairagon+N is the most specific; ENSEMBL is intermediate except in exact exon specificity, where it falls below the other two. None of the programs completely dominates any other, although one might argue that Pairagon+N has a slight edge, since the margin by which its specificity exceeds that of the second best program is substantially larger than the margin by which its sensitivity falls below the others. In absolute numbers, our pipeline identifies almost the same number of correct Gencode transcript structures as ENSEMBL (255 versus 258, respectively), and 21 fewer than ExoGean, but we have many fewer incorrect transcripts (149 versus 205 from ENSEMBL and 237 from ExoGean). Their gene accuracy measures are slightly better than ours because ENSEMBL and ExoGean predict more transcripts per gene locus on average. Predicting more transcripts at a locus increases the chance that at least one of them is correct, yielding a true positive by the gene measure, while no penalty in false positives is incurred for the additional incorrect transcripts. This is arguably a flaw in the gene level measure when applied to systems that can predict more than one transcript per locus.

### Pairagon's cDNA alignments are highly accurate

The individual accuracies of Pairagon and N-SCAN_EST gene structures in the submission are given in Table 2. Pairagon's nucleotide and exon specificities are 98.8% and 96.1%, respectively. Pairagon is also very accurate in identifying splice sites - we estimated that 98.3% of the introns that Pairagon identified have supporting evidence in the Gencode reference genes. When there is high quality mRNA evidence, more than three-fourths of transcript structures predicted by Pairagon are correct.

Identifying the correct splice boundaries is the crucial step in cDNA-to-genome alignment, and here Pairagon proves to be extremely accurate. Out of the 1,834 introns Pairagon predicted (both within and outside coding regions), only 22 introns from 15 transcript structures were not supported by HAVANA annotation. Three of them (from a single transcript) matched the introns of a Gencode gene labeled 'putative' and eight of them were a result of using incorrect seed exons from BLASTN (discussed in detail below). The remaining 11 were from Refseq cDNAs that have no evidence in HAVANA annotation. Two of the eleven aligned to the reference genome with numerous mismatches.

**Figure 3:**
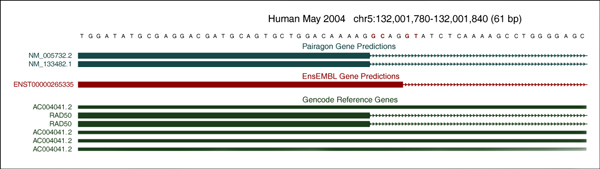
An annotated GC donor site that ENSEMBL misses. There is a GT dinucleotide four nucleotides downstream of the GC donor site (both dinucleotides are marked brown in the sequence). Pairagon identifies the correct donor site. (Screen shot obtained from UCSC Genome Browser web site [23].)

There are 22 unique GC-AG introns in the protein coding part of the HAVANA annotation. Pairagon correctly identifies 12 of these. The remaining 10 are missed because they did not have supporting Refseq or MGC cDNA sequence. When other systems prefer a GT dinucleotide, especially if it occurs close to the actual GC donor site, Pairagon gets the GC splice boundaries correct. Figure 3 shows one such example where ENSEMBL, Augustus and ExonHunter choose an incorrect GT donor site that is four nucleotides downstream of the correct GC donor, which Pairagon chooses. There are two unique AT-AC splice sites in the annotation and Pairagon correctly identifies both of them. Among the methods that use mRNA/EST evidence, AceView identifies the two introns and ENSEMBL identifies one of them. There are also two AT-AG introns with one supporting Gencode annotation each, and only AceView predicts them. Pairagon's splice boundary model prevents it from identifying these introns.

**Figure 4:**
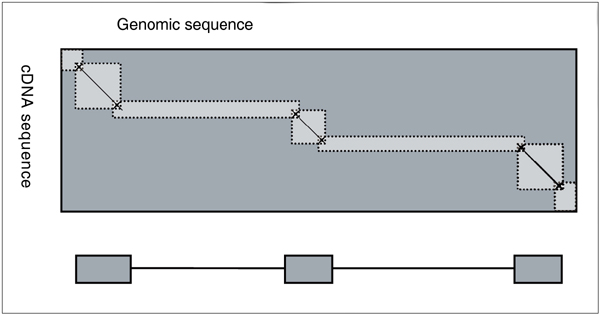
Generating the search subspace given three high-scoring segment pairs (HSPs) in the Stepping Stone algorithm. The three diagonal lines represent the three HSPs. The stars represent alignment pins. The lighter blue areas represent the search subspaces that are actually used in the heuristic method. The optimal algorithm uses the entire rectangle in blue. The block diagram shows the optimal spliced alignment where blue boxes represent an exon and the thin lines represent an intron.

**Figure 5:**
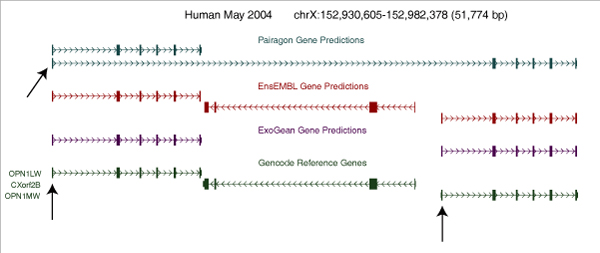
An incorrect alignment from Pairagon. The seed alignment from BLASTN aligned the 112-base exon at a location about 30 kb upstream (arrow in Pairagon gene prediction) instead of the annotated location (arrows in Gencode reference genes). Both alignments for that exon are 100% identical. (Screen shot obtained from UCSC Genome Browser web site [23].)

In the Stepping Stone implementation of Pairagon, the accuracy of the final alignment depends on how well the seed exons are mapped in the genome (see Materials and methods and Figure 4 for details). Figure 5 shows an example where the first 112 bases (forming an exon) of the cDNA can be mapped to either of two tandem duplicates that are identical in those 112 bases. Because we chose to use BLASTN parameter topComboN = 1, which does not return alignments of a query segment to more than one location in the genome, BLASTN aligned the exon arbitrarily to the locus farther from the rest of the alignment. As a result, Pairagon placed the exon in the same general region, while the annotation maps it to the nearer locus. One possible way to address this problem would be to follow Zhang and Gish [18], who report using topComboN = 4 to generate multiple combinations of high-scoring segment pairs (HSPs) as seed alignments for their cDNA-to-genome alignment program, EXALIN. We can then superimpose the search subspaces obtained from the possible HSP combinations. Using this approach, Pairagon would choose the correct alignment for the example in Figure 5 because, all other things being equal, it favors shorter introns over longer ones.

### Pairagon's accuracy has improved since the official evaluation

Since the EGASP assessment, we have made several improvements to both Pairagon's probability model and its implementation. We have retrained Pairagon using its own alignments of 20,594 MGC cDNA sequences to 21,249 loci on the human genome. Several bug-fixes and optimizations have resulted in a faster and more robust program with lower memory requirements. Table 3 lists the accuracy measures of the current version of Pairagon (v0.95) when aligning the same cDNA sequences used for the assessment. Pairagon v0.95 shows improvement in all accuracy measures. It now identifies 22 more correct Gencode transcripts and 162 more correct exons with a small improvement in specificity as well. Thus, the accuracy of our pipeline using Pairagon v0.95 is substantially better than that of the version submitted for the assessment, which was already as good as, or slightly better than, that of the other entrants. Of course, other systems have likely improved as a result of this exercise, too.

### A lack of biological evidence raises questions about ORF annotation

Identifying the coding region in (even) a full-length mRNA is an extremely difficult problem. NCBI and HAVANA do not always agree in their CDS annotations of mRNA sequences, even if they agree on the exon-intron structures. Because we relied on the CDS annotations from NCBI, a few of our gene predictions are incorrect according to HAVANA, although the underlying alignment is correct. For example, GenBank's annotated translation start sites for cDNA sequences BC001940 and NM_001004759.1 are 798 bases downstream and 81 bases upstream of HAVANA's annotated translation start sites in Gencode genes AC005538.1-001 and AC011711.3-001, respectively. A few more of our ORF predictions obtained from correct alignments are labeled incorrect because HAVANA has not made any CDS annotations on the exon-intron structures yet. For example, the exon-intron structure of our gene NM_181879.1 from aligning a reviewed RefSeq mRNA NM_181879.1 matches that of Gencode reference gene AC008984.1-003, which does not have a CDS annotation. Since the biological evidence supporting the GenBank ORF annotations, if any, is not available for evaluation, we might do better by using a modified version of N-SCAN to predict ORFs on aligned cDNA sequences.

### N-SCAN_EST performs well on complete GENCODE test regions

After the release of the HAVANA annotations, we found that N-SCAN_EST predictions used to fill the gaps between Pairagon alignments had a very high proportion of incorrect genes - the gene/transcript specificity of the original N-SCAN_EST predictions was 8.5% in regions that did not overlap Pairagon alignments (gene and transcript specificity are the same for programs that predict only one transcript per locus). However, this is due largely to the fact that there are high quality cDNA sequences covering most of the real genes in the ENCODE regions. When these are not used and N-SCAN_EST's predictions on the complete GENCODE test regions are evaluated, their specificity is 38.7% (Table 2).

In the ENCODE regions, the accuracy of N-SCAN_EST is due in large part to the accuracy of N-SCAN itself (this may not hold in less gene-dense regions). Table 4 compares the five submissions to the Dual or Multiple Genome category of EGASP that score the highest on exons, transcripts, and genes. N-SCAN scores the highest in all categories except for nucleotide sensitivity. In terms of exon specificity, N-SCAN is 4.8% better than the next best system (Dogfish) and in transcript specificity 18% better than the next best system (Augustus-dual). For transcript and exon sensitivity, N-SCAN is 4.7% and 4.6% better, respectively, than any other system except TWINSCAN-MARS. N-SCAN outperforms TWINSCAN-MARS by about 1% transcript sensitivity and 2% exon sensitivity. TWINSCAN-MARS has relatively high sensitivity in part because it predicts several transcripts per gene, for which it pays a price in specificity. Even with the hit it takes in specificity, TWINSCAN-MARS is among the top three performers, especially at the transcript level. This may be explained, in part, by the fact that N-SCAN and TWINSCAN-MARS share nearly identical models for DNA sequence [16], although their conservation models are quite different.

**Figure 6:**
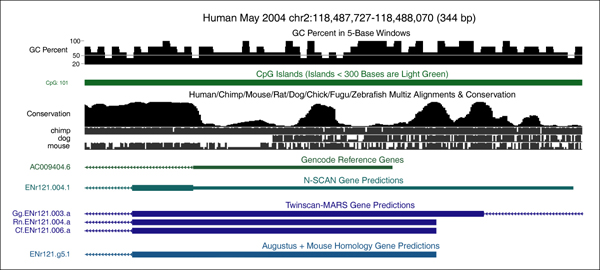
Initial exon of a gene where N-SCAN correctly discriminates coding region from the 5' UTR. Other gene prediction systems predict longer coding regions due to the high G+C content of the region. (Screen shot obtained from UCSC Genome Browser web site [23].)

N-SCAN's ability to explicitly model untranslated regions (UTRs) [12,13,19] facilitates the distinction between coding and non-coding exons. Figure 6 illustrates this advantage of N-SCAN when compared to other dual- or multiple-genome gene predictors on Gencode reference gene AC009404.6. Only the N-SCAN prediction agrees with the Gencode reference gene; N-SCAN's ability to model 5' UTR content is the key. The 168 base-pair (bp) region upstream of the annotated start codon lies within a 1,012 bp CpG island (annotated on the UCSC Genome Browser CpG-island track). The 67% G+C content of this 168 bp region is very high compared to typical intronic and intergenic regions and even high compared to most exonic regions. However, this is not unusual for a region of this size within a CpG island. Without explicit 5' UTR-content modeling, however, it is more likely to be predicted as a coding region rather than as a 5' UTR, intronic, or intergenic region. For example, Augustus + Mouse Homology and TWINSCAN-MARS annotate this region as coding. N-SCAN's modeling of DNA content and conserved sequence for 5' UTR regions facilitates the correct categorization of this region.

**Figure 7:**
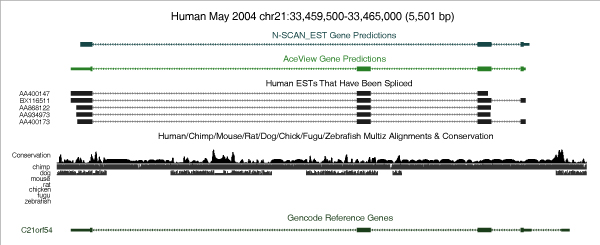
A gene where N-SCAN_EST predicts three out of the four exons right. All other programs except AceView do not predict anything in that locus. N-SCAN_EST missed an exon even though there is EST evidence for it. We believe that lack of conservation overwhelmed the EST evidence for that exon. (Screen shot obtained from UCSC Genome Browser web site [23].)

When the genome sequence and conservation do not provide sufficient information about the coding potential of a gene locus, EST evidence can be very useful in gene prediction. Figure 7 shows a gene where N-SCAN_EST predicts three out of four exons correctly while both ENSEMBL and N-SCAN do not predict any gene in the region. In fact, N-SCAN_EST is one of only two gene predictors that predict any gene in this locus. There are high quality EST alignments supporting this gene, such as BX116511 with a 100% identical alignment of 583 bases, which aid N-SCAN_EST in predicting this gene even though the conservation rate of the coding regions is low. This low conservation may explain why N-SCAN failed to predict a gene; likewise, the extremely low genomic conservation in Exon 3 may explain why even N-SCAN_EST missed this exon.

## Conclusion

The results of this exercise have demonstrated two things. First, this careful community assessment has been very valuable, particularly for the way in which it uncovered weaknesses in, and inspired improvements to, Pairagon and other systems. Second, genome annotation without trans alignments can compete successfully with systems like ENSEMBL and ExoGean, which use *trans *alignments, under certain circumstances. However, annotation accuracy in the EGASP assessment is determined largely by the accuracy with which high quality native cDNA sequences can be aligned, and secondarily by the accuracy with which HAVANA's ORF calls on those cDNA sequences, or lack thereof, can be anticipated. We cannot extrapolate the results of this exercise to situations in which fewer full length cDNAs and/or fewer ESTs are available. In such situations, the accuracy of our pipeline would depend more on N-SCAN and N-SCAN_EST, while the accuracy of ENSEMBL would depend more on *trans *alignments. In future assessments, it would be worthwhile to assess prediction pipelines under a range of scenarios between the two evaluated this time - freedom to use all available native cDNA and prohibition against using any. In particular, the selective elimination of cDNA and EST sequences from the available pool would shed light on the tradeoffs among different approaches under a range of situations of practical significance (see [4] for such a study on Pairagon+N-SCAN_EST).

## Materials and methods

### Pairagon gene predictions

The state diagram of Pairagon's pairHMM model for cDNA-to-genome alignment is given in Figure 1. The different states model different alignment columns as follows: matches and mismatches are modeled by state A; intron is modeled by a loop consisting of Entry, Intron and Exit; insertion and gap in genome are modeled by states G and C, respectively. Four additional states - RG1, RC1, RG2 and RC2 modeling unaligned genomic and cDNA sequences - were added to facilitate local alignment. Although, for simplicity, Figure 1 shows only one loop modeling introns, our model contains two such loops. One of them requires GT or GC at the splice donor site and AG at the splice acceptor site. The other requires AT and AC at those sites, respectively. Each state can emit the different columns of a cDNA-to-genome alignment with certain probabilities (emission probabilities). For each state there is also a probability of staying in that state or transitioning to different states (transition probabilities). These probabilities can be estimated using maximum likelihood from example alignments.

We implemented the Viterbi algorithm, an optimal dynamic programming algorithm for finding the most probable alignment between two sequences, in C. Although it produced accurate alignments, the time and space complexity for optimally aligning two sequences increases in proportion to the product of the sizes of the input sequences, imposing limitations on the size of the input sequences. Therefore, we adapted the Stepping Stone algorithm [20], a heuristic modification to the optimal algorithm. Stepping Stone relies on faster seeded alignment programs like BLASTN to identify regions of high identity between the cDNA and the genomic sequence (diagonal lines in Figure 4). It restricts the optimal dynamic programming algorithm to regions close to the approximate exons that the seed alignments correspond to (light blue region in Figure 4).

Pairagon v0.5 was trained using 15,766 BLAT alignments of 15,297 MGC [1,9,10] cDNA sequences to the human genome build NCBI35 (May 2005). Transition probabilities between the states were estimated from the alignments using maximum likelihood. Because this was a bootstrap procedure, and BLAT does not pay careful attention to splice sites, we assigned reasonable estimates for probabilities of GT-AG, GC-AG and AT-AC splice site combinations (98.9%, 1.0% and 0.1%, respectively). All bases were equally probable in states RG1, RC1, RG2, RC2, G, C and Intron. The probability of a match in the aligned state was estimated using maximum likelihood and was evenly distributed among the four possible combinations. Similarly, the probability of a mismatch in the aligned state was distributed among the 12 possible combinations.

Ungapped local alignments between the cDNA sequences and the unmasked ENCODE regions were generated using BLASTN [21] with parameters M = 1 N = -3. These approximate seed exons were then used by the Stepping Stone implementation of Pairagon v0.5 to generate an alignment. GenBank CDS annotations of the cDNA sequences were used to convert these alignments into gene structures.

### N-SCAN gene predictions

The genome sequence was masked for putative processed pseudogenes using PPFINDER [5]. N-SCAN gene predictions were then obtained as explained in [12,13].

### N-SCAN_EST gene predictions

Human ESTs, downloaded from dbEST on 20 January 2005, were aligned to whole human genome (build NCBI35) by BLAT [8]. For each EST sequence, the alignment with the greatest number of bases matching the genome was selected. Alignments with at least 98% of the bases in the entire EST matching the genome were chosen to generate an ESTseq for each chromosome. ESTseq parameters were estimated from regions corresponding to a set of cleaned Refseq annotations containing 17,798 transcripts. An additional 1,000 bases on either side of the genes were used to train intergenic regions. The genome sequence was masked for putative processed pseudogenes using PPFINDER [5]. ESTseqs corresponding to the ENCODE regions were obtained by cutting the relevant sections out of the chromosomal ESTseq, and N-SCAN_EST was then used to predict genes.

### Pairagon+N-SCAN_EST pipeline

A block diagram showing the steps involved in generating Pairagon gene structures and N-SCAN_EST gene predictions, and combining them is given in Figure 2. Because multiple mRNA sequences are available for some genes, identical Pairagon gene structures are merged into one gene. N-SCAN_EST predictions are added to the final set if they do not overlap the merged Pairagon gene structures. We used the Eval software package [22] for finding these overlapping genes.

## Figures and Tables

**Table 1:** Prediction accuracy measures of mRNA/EST evidence based gene prediction methods

Name	NSn	NSp	ESn	ESp	ExT	TSn	TSp	TrG	GSn	GSp
Pairagon + N-SCAN_EST	87.6	92.8	76.6	**89.0**	7.2	39.3	**60.6**	1.3	69.6	61.7
ENSEMBL	**90.2**	92.0	77.5	82.7	7.8	39.8	54.6	1.5	**71.6**	67.3
ExoGean	84.2	**94.3**	**79.3**	83.5	9.8	**42.5**	52.4	2.3	63.2	**80.8**

The highest value for each measure is in bold. The columns are Nucleotide sensitivity (NSn) and specificity (NSp), Exon sensitivity (ESn) and specificity (ESp), Exons per transcript (ExT), Transcript sensitivity (TSn) and specificity (TSp), Transcripts per gene (TrG), and Gene sensitivity (GSn) and specificity (GSp).

**Table 2:** Individual prediction accuracies of Pairagon alignments and N-SCAN_EST predictions in the submission

Name	NSn	NSp	ESn	ESp	ExT	TSn	TSp	TrG	GSn	GSp
Pairagon	71.5	98.8	66.8	96.1	8.1	37.9	76.5	1.4	66.9	84.2
N-SCAN_EST	84.9	91.1	72.2	84.5	8.1	18.3	38.7	1.0	38.2	38.7
N-SCAN_EST*	16.0	73.0	9.8	59.1	4.1	1.4	8.5	1.0	2.7	8.5
N-SCAN_EST^†^	72.2	73.0	57.6	59.1	4.1	8.3	8.5	1.0	12.7	8.5

*N-SCAN_EST predictions not overlapping Pairagon alignments (sensitivities measured against all Gencode genes). ^†^N-SCAN_EST predictions not overlapping Pairagon alignments (sensitivities measured against Gencode genes not overlapping Pairagon alignments). Columns are defined as in Table 1.

**Table 3:** Prediction accuracies of improved Pairagon alignments and Pairagon+N-SCAN_EST gene structures

Name	NSn	NSp	ESn	ESp	ExT	TSn	TSp	TrG	GSn	GSp
Pairagon v0.95	78.8	99.2	72.7	96.5	8.4	41.3	77.0	1.4	71.3	84.3
Pairagon v0.0.95 + N-SCAN_EST	89.9	92.5	79.0	88.9	7.6	42.4	63.3	1.3	73.3	64.5

Columns are defined as in Table 1.

**Table 4:** Prediction accuracy measures of multiple-genome based gene prediction methods

Name	NSn	NSp	ESn	ESp	ExT	TSn	TSp	TrG	GSn	GSp
Augustus-dual	**88.9**	80.2	63.1	69.1	6.1	12.3	18.6	1.0	26.0	18.6
N-SCAN	85.4	**89.0**	**67.7**	**82.1**	8.0	**17.0**	**36.7**	1.0	**35.5**	**36.7**
Twinscan-MARS	84.3	74.1	65.6	61.7	8.6	15.9	15.1	1.7	33.5	24.9
Saga	52.5	81.4	38.8	50.7	5.6	2.2	3.4	1.0	4.4	3.4
Dogfish	64.8	88.2	53.1	77.3	8.7	5.1	14.6	1.0	10.8	14.6

The highest value for each measure is in bold. Columns are defined as in Table 1.
